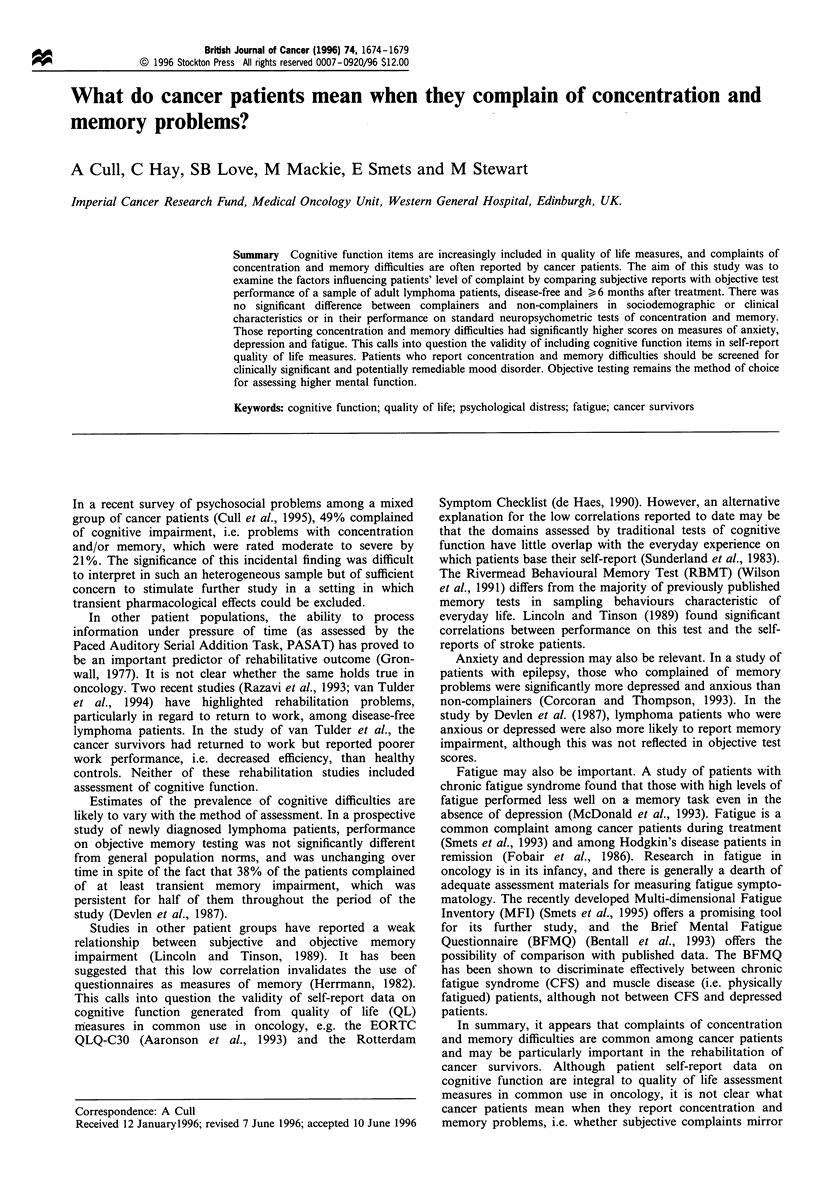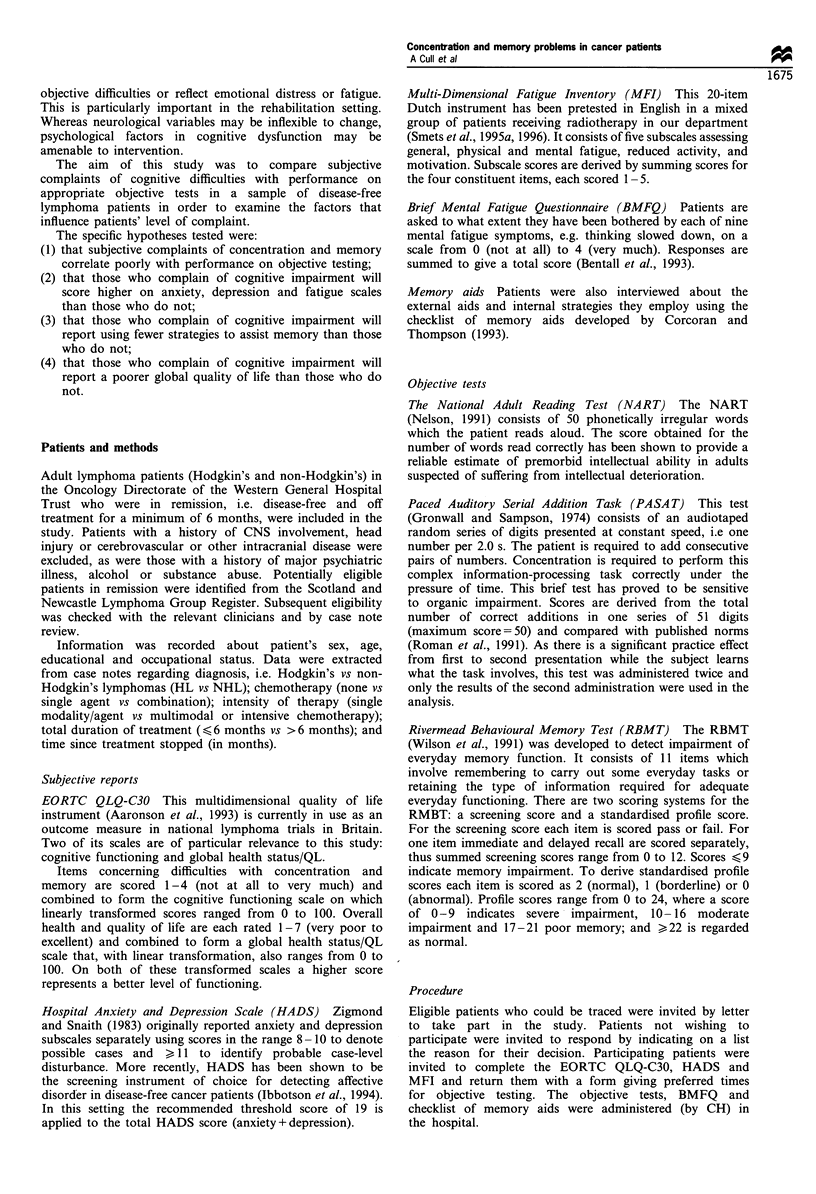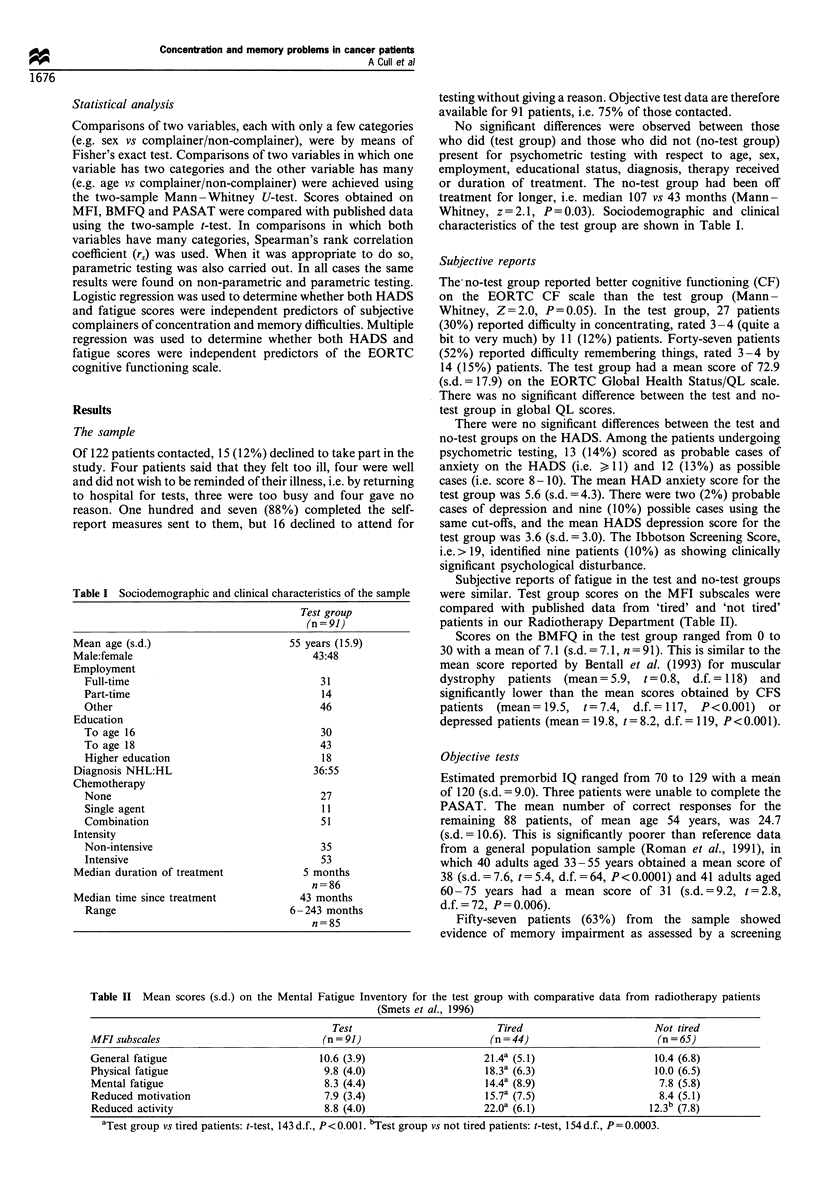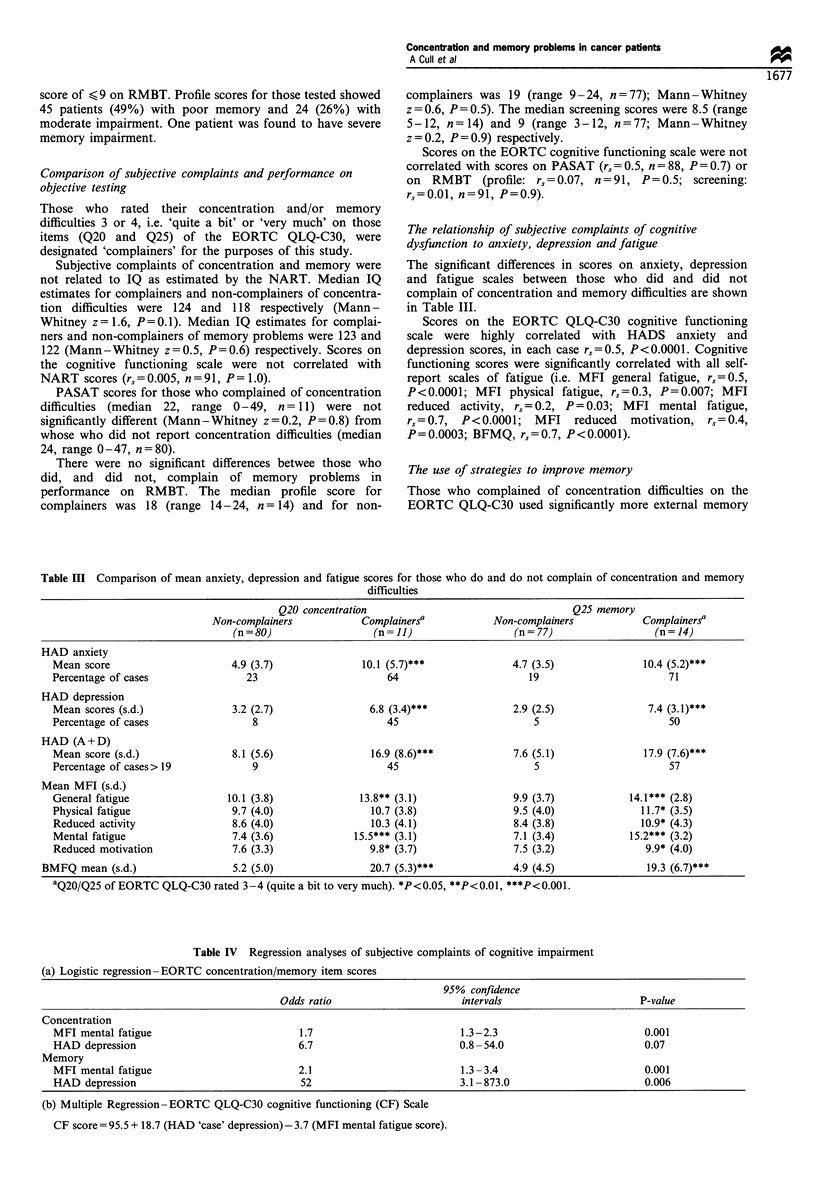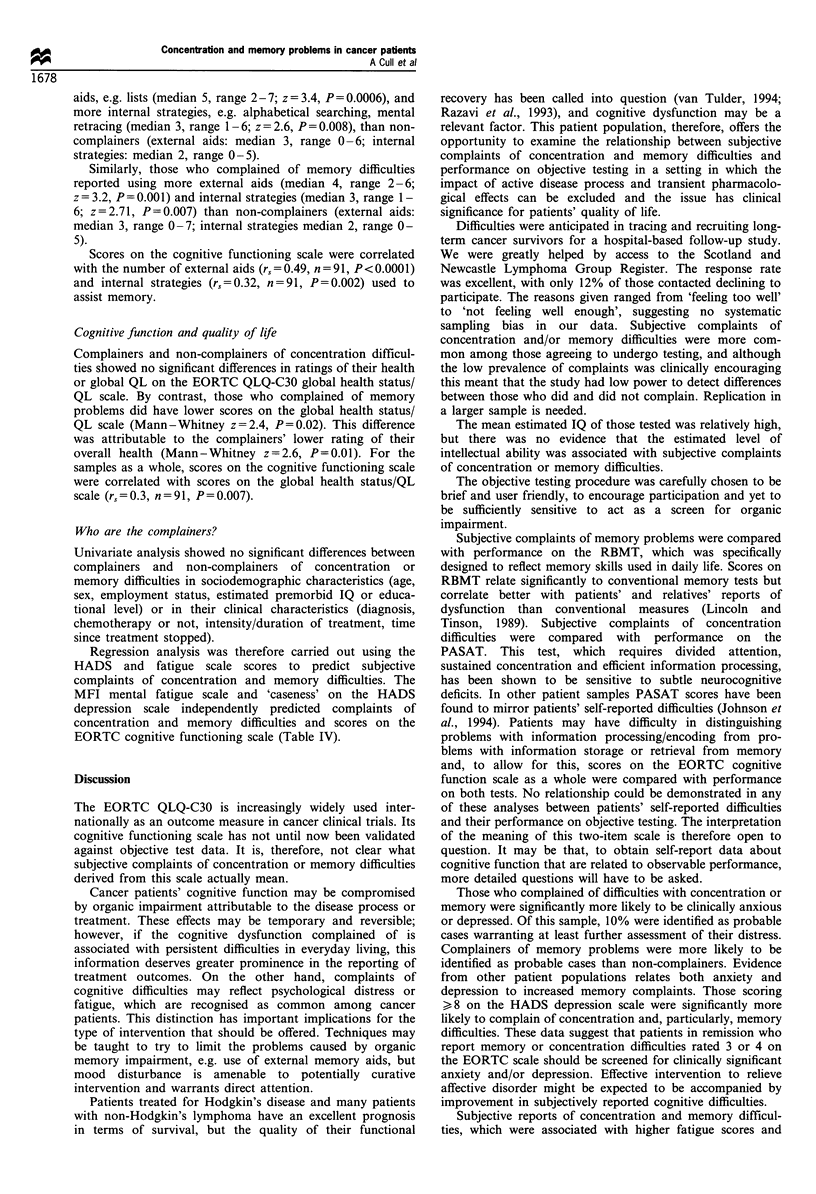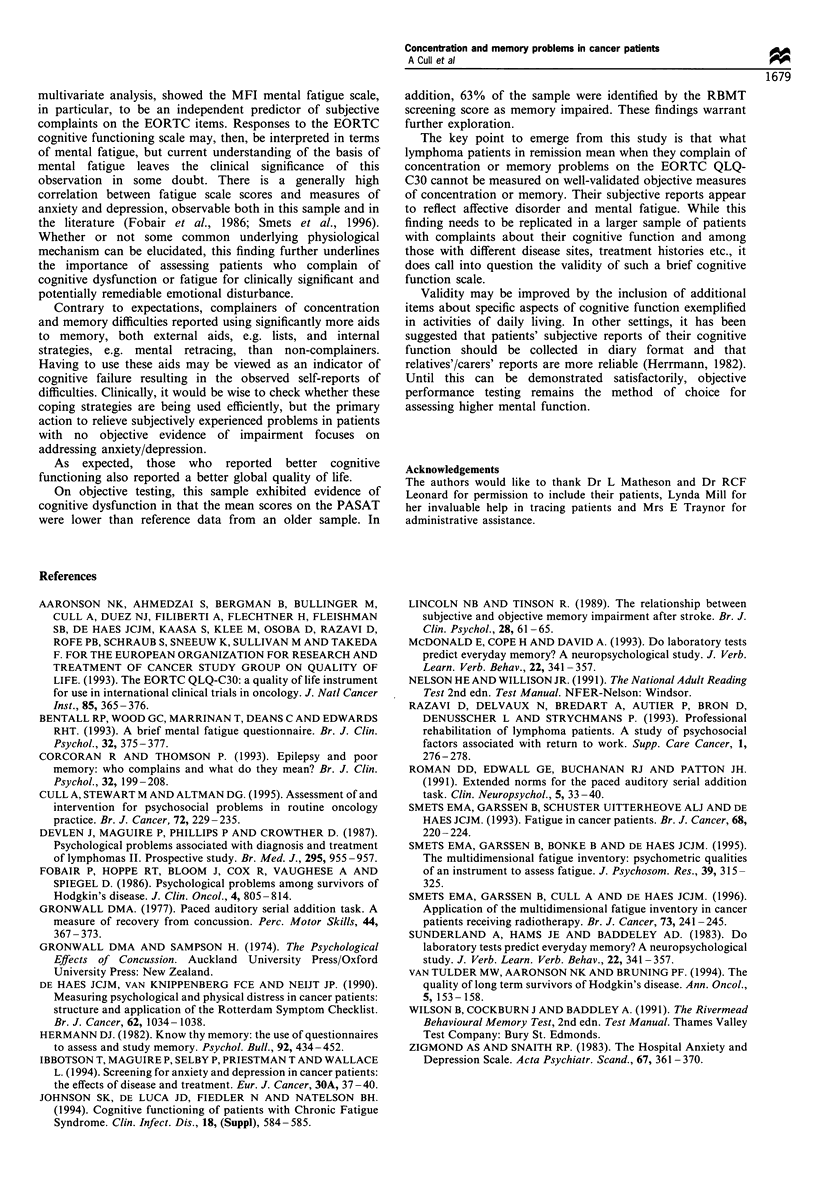# What do cancer patients mean when they complain of concentration and memory problems?

**DOI:** 10.1038/bjc.1996.608

**Published:** 1996-11

**Authors:** A. Cull, C. Hay, S. B. Love, M. Mackie, E. Smets, M. Stewart

**Affiliations:** Imperial Cancer Research Fund, Medical Oncology Unit, Western General Hospital, Edinburgh, UK.

## Abstract

Cognitive function items are increasingly included in quality of life measures, and complaints of concentration and memory difficulties are often reported by cancer patients. The aim of this study was to examine the factors influencing patients' level of complaint by comparing subjective reports with objective test performance of a sample of adult lymphoma patients, disease-free and > or = 6 months after treatment. There was no significant difference between complainers and non-complainers in sociodemographic or clinical characteristics or in their performance on standard neuropsychometric tests of concentration and memory. Those reporting concentration and memory difficulties had significantly higher scores on measures of anxiety, depression and fatigue. This calls into question the validity of including cognitive function items in self-report quality of life measures. Patients who report concentration and memory difficulties should be screened for clinically significant and potentially remediable mood disorder. Objective testing remains the method of choice for assessing higher mental function.


British Journal of Cancer (1996) 74, 1674-1679
© 1996 Stockton Press All rights reserved 0007-0920/96 $12.00

### What do cancer patients mean when they complain of concentration and memory problems?

A Cull, C Hay, SB Love, M Mackie, E Smets and M Stewart

*Imperial Cancer Research Fund, Medical Oncology Unit, Western General Hospital, Edinburgh, UK.*

**Summary** Cognitive function items are increasingly included in quality of life measures, and complaints of concentration and memory difficulties are often reported by cancer patients. The aim of this study was to examine the factors influencing patients' level of complaint by comparing subjective reports with objective test performance of a sample of adult lymphoma patients, disease-free and ≥6 months after treatment. There was no significant difference between complainers and non-complainers in sociodemographic or clinical characteristics or in their performance on standard neuropsychometric tests of concentration and memory. Those reporting concentration and memory difficulties had significantly higher scores on measures of anxiety, depression and fatigue. This calls into question the validity of including cognitive function items in self-report quality of life measures. Patients who report concentration and memory difficulties should be screened for clinically significant and potentially remediable mood disorder. Objective testing remains the method of choice for assessing higher mental function.

**Keywords:** cognitive function; quality of life; psychological distress; fatigue; cancer survivors

In a recent survey of psychosocial problems among a mixed group of cancer patients (Cull *et al.*, 1995), 49% complained of cognitive impairment, i.e. problems with concentration and/or memory, which were rated moderate to severe by 21%. The significance of this incidental finding was difficult to interpret in such an heterogeneous sample but of sufficient concern to stimulate further study in a setting in which transient pharmacological effects could be excluded.

In other patient populations, the ability to process information under pressure of time (as assessed by the Paced Auditory Serial Addition Task, PASAT) has proved to be an important predictor of rehabilitative outcome (Gronwall, 1977). It is not clear whether the same holds true in oncology. Two recent studies (Razavi *et al.*, 1993; van Tulder *et al.*, 1994) have highlighted rehabilitation problems, particularly in regard to return to work, among disease-free lymphoma patients. In the study of van Tulder *et al.*, the cancer survivors had returned to work but reported poorer work performance, i.e. decreased efficiency, than healthy controls. Neither of these rehabilitation studies included assessment of cognitive function.

Estimates of the prevalence of cognitive difficulties are likely to vary with the method of assessment. In a prospective study of newly diagnosed lymphoma patients, performance on objective memory testing was not significantly different from general population norms, and was unchanging over time in spite of the fact that 38% of the patients complained of at least transient memory impairment, which was persistent for half of them throughout the period of the study (Devlen *et al.*, 1987).

Studies in other patient groups have reported a weak relationship between subjective and objective memory impairment (Lincoln and Tinson, 1989). It has been suggested that this low correlation invalidates the use of questionnaires as measures of memory (Herrmann, 1982). This calls into question the validity of self-report data on cognitive function generated from quality of life (QL) measures in common use in oncology, e.g. the EORTC QLQ-C30 (Aaronson *et al.*, 1993) and the Rotterdam Symptom Checklist (de Haes, 1990). However, an alternative explanation for the low correlations reported to date may be that the domains assessed by traditional tests of cognitive function have little overlap with the everyday experience on which patients base their self-report (Sunderland *et al.*, 1983). The Rivermead Behavioural Memory Test (RBMT) (Wilson *et al.*, 1991) differs from the majority of previously published memory tests in sampling behaviours characteristic of everyday life. Lincoln and Tinson (1989) found significant correlations between performance on this test and the self-reports of stroke patients.

Anxiety and depression may also be relevant. In a study of patients with epilepsy, those who complained of memory problems were significantly more depressed and anxious than non-complainers (Corcoran and Thompson, 1993). In the study by Devlen *et al.* (1987), lymphoma patients who were anxious or depressed were also more likely to report memory impairment, although this was not reflected in objective test scores.

Fatigue may also be important. A study of patients with chronic fatigue syndrome found that those with high levels of fatigue performed less well on a memory task even in the absence of depression (McDonald *et al.*, 1993). Fatigue is a common complaint among cancer patients during treatment (Smets *et al.*, 1993) and among Hodgkin's disease patients in remission (Fobair *et al.*, 1986). Research in fatigue in oncology is in its infancy, and there is generally a dearth of adequate assessment materials for measuring fatigue symptomatology. The recently developed Multi-dimensional Fatigue Inventory (MFI) (Smets *et al.*, 1995) offers a promising tool for its further study, and the Brief Mental Fatigue Questionnaire (BFMQ) (Bentall *et al.*, 1993) offers the possibility of comparison with published data. The BFMQ has been shown to discriminate effectively between chronic fatigue syndrome (CFS) and muscle disease (i.e. physically fatigued) patients, although not between CFS and depressed patients.

In summary, it appears that complaints of concentration and memory difficulties are common among cancer patients and may be particularly important in the rehabilitation of cancer survivors. Although patient self-report data on cognitive function are integral to quality of life assessment measures in common use in oncology, it is not clear what cancer patients mean when they report concentration and memory problems, i.e. whether subjective complaints mirror

Correspondence: A Cull
Received 12 January1996; revised 7 June 1996; accepted 10 June 1996

objective difficulties or reflect emotional distress or fatigue. This is particularly important in the rehabilitation setting. Whereas neurological variables may be inflexible to change, psychological factors in cognitive dysfunction may be amenable to intervention.

The aim of this study was to compare subjective complaints of cognitive difficulties with performance on appropriate objective tests in a sample of disease-free lymphoma patients in order to examine the factors that influence patients' level of complaint.

The specific hypotheses tested were:

(1) that subjective complaints of concentration and memory correlate poorly with performance on objective testing;
(2) that those who complain of cognitive impairment will score higher on anxiety, depression and fatigue scales than those who do not;
(3) that those who complain of cognitive impairment will report using fewer strategies to assist memory than those who do not;
(4) that those who complain of cognitive impairment will report a poorer global quality of life than those who do not.

### Patients and methods

Adult lymphoma patients (Hodgkin's and non-Hodgkin's) in the Oncology Directorate of the Western General Hospital Trust who were in remission, i.e. disease-free and off treatment for a minimum of 6 months, were included in the study. Patients with a history of CNS involvement, head injury or cerebrovascular or other intracranial disease were excluded, as were those with a history of major psychiatric illness, alcohol or substance abuse. Potentially eligible patients in remission were identified from the Scotland and Newcastle Lymphoma Group Register. Subsequent eligibility was checked with the relevant clinicians and by case note review.

Information was recorded about patient's sex, age, educational and occupational status. Data were extracted from case notes regarding diagnosis, i.e. Hodgkin's *vs* non-Hodgkin's lymphomas (HL *vs* NHL); chemotherapy (none *vs* single agent *vs* combination); intensity of therapy (single modality/agent *vs* multimodal or intensive chemotherapy); total duration of treatment ($\leqslant$6 months *vs* >6 months); and time since treatment stopped (in months).

### *Subjective reports*

*EORTC QLQ-C30* This multidimensional quality of life instrument (Aaronson *et al.*, 1993) is currently in use as an outcome measure in national lymphoma trials in Britain. Two of its scales are of particular relevance to this study: cognitive functioning and global health status/QL.

Items concerning difficulties with concentration and memory are scored 1–4 (not at all to very much) and combined to form the cognitive functioning scale on which linearly transformed scores ranged from 0 to 100. Overall health and quality of life are each rated 1–7 (very poor to excellent) and combined to form a global health status/QL scale that, with linear transformation, also ranges from 0 to 100. On both of these transformed scales a higher score represents a better level of functioning.

*Hospital Anxiety and Depression Scale (HADS)* Zigmond and Snaith (1983) originally reported anxiety and depression subscales separately using scores in the range 8–10 to denote possible cases and $\geqslant$11 to identify probable case-level disturbance. More recently, HADS has been shown to be the screening instrument of choice for detecting affective disorder in disease-free cancer patients (Ibbotson *et al.*, 1994). In this setting the recommended threshold score of 19 is applied to the total HADS score (anxiety + depression).

*Multi-Dimensional Fatigue Inventory (MFI)* This 20-item Dutch instrument has been pretested in English in a mixed group of patients receiving radiotherapy in our department (Smets *et al.*, 1995*a*, 1996). It consists of five subscales assessing general, physical and mental fatigue, reduced activity, and motivation. Subscale scores are derived by summing scores for the four constituent items, each scored 1–5.

*Brief Mental Fatigue Questionnaire (BMFQ)* Patients are asked to what extent they have been bothered by each of nine mental fatigue symptoms, e.g. thinking slowed down, on a scale from 0 (not at all) to 4 (very much). Responses are summed to give a total score (Bentall *et al.*, 1993).

*Memory aids* Patients were also interviewed about the external aids and internal strategies they employ using the checklist of memory aids developed by Corcoran and Thompson (1993).

### *Objective tests*

*The National Adult Reading Test (NART)* The NART (Nelson, 1991) consists of 50 phonetically irregular words which the patient reads aloud. The score obtained for the number of words read correctly has been shown to provide a reliable estimate of premorbid intellectual ability in adults suspected of suffering from intellectual deterioration.

*Paced Auditory Serial Addition Task (PASAT)* This test (Gronwall and Sampson, 1974) consists of an audiotaped random series of digits presented at constant speed, i.e one number per 2.0 s. The patient is required to add consecutive pairs of numbers. Concentration is required to perform this complex information-processing task correctly under the pressure of time. This brief test has proved to be sensitive to organic impairment. Scores are derived from the total number of correct additions in one series of 51 digits (maximum score = 50) and compared with published norms (Roman *et al.*, 1991). As there is a significant practice effect from first to second presentation while the subject learns what the task involves, this test was administered twice and only the results of the second administration were used in the analysis.

*Rivermead Behavioural Memory Test (RBMT)* The RBMT (Wilson *et al.*, 1991) was developed to detect impairment of everyday memory function. It consists of 11 items which involve remembering to carry out some everyday tasks or retaining the type of information required for adequate everyday functioning. There are two scoring systems for the RMBT: a screening score and a standardised profile score. For the screening score each item is scored pass or fail. For one item immediate and delayed recall are scored separately, thus summed screening scores range from 0 to 12. Scores $\leqslant$9 indicate memory impairment. To derive standardised profile scores each item is scored as 2 (normal), 1 (borderline) or 0 (abnormal). Profile scores range from 0 to 24, where a score of 0–9 indicates severe impairment, 10–16 moderate impairment and 17–21 poor memory; and $\geqslant$22 is regarded as normal.

### *Procedure*

Eligible patients who could be traced were invited by letter to take part in the study. Patients not wishing to participate were invited to respond by indicating on a list the reason for their decision. Participating patients were invited to complete the EORTC QLQ-C30, HADS and MFI and return them with a form giving preferred times for objective testing. The objective tests, BMFQ and checklist of memory aids were administered (by CH) in the hospital.

### Statistical analysis

Comparisons of two variables, each with only a few categories (e.g. sex vs complainer/non-complainer), were by means of Fisher's exact test. Comparisons of two variables in which one variable has two categories and the other variable has many (e.g. age vs complainer/non-complainer) were achieved using the two-sample Mann–Whitney U-test. Scores obtained on MFI, BMFQ and PASAT were compared with published data using the two-sample t-test. In comparisons in which both variables have many categories, Spearman's rank correlation coefficient ($r_s$) was used. When it was appropriate to do so, parametric testing was also carried out. In all cases the same results were found on non-parametric and parametric testing. Logistic regression was used to determine whether both HADS and fatigue scores were independent predictors of subjective complainers of concentration and memory difficulties. Multiple regression was used to determine whether both HADS and fatigue scores were independent predictors of the EORTC cognitive functioning scale.

### Results

### The sample

Of 122 patients contacted, 15 (12%) declined to take part in the study. Four patients said that they felt too ill, four were well and did not wish to be reminded of their illness, i.e. by returning to hospital for tests, three were too busy and four gave no reason. One hundred and seven (88%) completed the self-report measures sent to them, but 16 declined to attend for testing without giving a reason. Objective test data are therefore available for 91 patients, i.e. 75% of those contacted.

No significant differences were observed between those who did (test group) and those who did not (no-test group) present for psychometric testing with respect to age, sex, employment, educational status, diagnosis, therapy received or duration of treatment. The no-test group had been off treatment for longer, i.e. median 107 vs 43 months (Mann–Whitney, $z=2.1$, $P=0.03$). Sociodemographic and clinical characteristics of the test group are shown in Table I.

**Table I** Sociodemographic and clinical characteristics of the sample

| | Test group (n=91) |
|---|---|
| Mean age (s.d.) | 55 years (15.9) |
| Male:female | 43:48 |
| Employment | |
| Full-time | 31 |
| Part-time | 14 |
| Other | 46 |
| Education | |
| To age 16 | 30 |
| To age 18 | 43 |
| Higher education | 18 |
| Diagnosis NHL:HL | 36:55 |
| Chemotherapy | |
| None | 27 |
| Single agent | 11 |
| Combination | 51 |
| Intensity | |
| Non-intensive | 35 |
| Intensive | 53 |
| Median duration of treatment | 5 months n=86 |
| Median time since treatment | 43 months |
| Range | 6–243 months n=85 |

### Subjective reports

The no-test group reported better cognitive functioning (CF) on the EORTC CF scale than the test group (Mann–Whitney, $Z=2.0$, $P=0.05$). In the test group, 27 patients (30%) reported difficulty in concentrating, rated 3–4 (quite a bit to very much) by 11 (12%) patients. Forty-seven patients (52%) reported difficulty remembering things, rated 3–4 by 14 (15%) patients. The test group had a mean score of 72.9 (s.d. = 17.9) on the EORTC Global Health Status/QL scale. There was no significant difference between the test and no-test group in global QL scores.

There were no significant differences between the test and no-test groups on the HADS. Among the patients undergoing psychometric testing, 13 (14%) scored as probable cases of anxiety on the HADS (i.e. $\geqslant 11$) and 12 (13%) as possible cases (i.e. score 8–10). The mean HAD anxiety score for the test group was 5.6 (s.d. = 4.3). There were two (2%) probable cases of depression and nine (10%) possible cases using the same cut-offs, and the mean HADS depression score for the test group was 3.6 (s.d. = 3.0). The Ibbotson Screening Score, i.e. > 19, identified nine patients (10%) as showing clinically significant psychological disturbance.

Subjective reports of fatigue in the test and no-test groups were similar. Test group scores on the MFI subscales were compared with published data from 'tired' and 'not tired' patients in our Radiotherapy Department (Table II).

Scores on the BMFQ in the test group ranged from 0 to 30 with a mean of 7.1 (s.d. = 7.1, $n=91$). This is similar to the mean score reported by Bentall et al. (1993) for muscular dystrophy patients (mean = 5.9, $t=0.8$, d.f. = 118) and significantly lower than the mean scores obtained by CFS patients (mean = 19.5, $t=7.4$, d.f. = 117, $P<0.001$) or depressed patients (mean = 19.8, $t=8.2$, d.f. = 119, $P<0.001$).

### Objective tests

Estimated premorbid IQ ranged from 70 to 129 with a mean of 120 (s.d. = 9.0). Three patients were unable to complete the PASAT. The mean number of correct responses for the remaining 88 patients, of mean age 54 years, was 24.7 (s.d. = 10.6). This is significantly poorer than reference data from a general population sample (Roman et al., 1991), in which 40 adults aged 33–55 years obtained a mean score of 38 (s.d. = 7.6, $t=5.4$, d.f. = 64, $P<0.0001$) and 41 adults aged 60–75 years had a mean score of 31 (s.d. = 9.2, $t=2.8$, d.f. = 72, $P=0.006$).

Fifty-seven patients (63%) from the sample showed evidence of memory impairment as assessed by a screening

**Table II** Mean scores (s.d.) on the Mental Fatigue Inventory for the test group with comparative data from radiotherapy patients (Smets et al., 1996)

| MFI subscales | Test (n=91) | Tired (n=44) | Not tired (n=65) |
|---|---|---|---|
| General fatigue | 10.6 (3.9) | 21.4[a] (5.1) | 10.4 (6.8) |
| Physical fatigue | 9.8 (4.0) | 18.3[a] (6.3) | 10.0 (6.5) |
| Mental fatigue | 8.3 (4.4) | 14.4[a] (8.9) | 7.8 (5.8) |
| Reduced motivation | 7.9 (3.4) | 15.7[a] (7.5) | 8.4 (5.1) |
| Reduced activity | 8.8 (4.0) | 22.0[a] (6.1) | 12.3[b] (7.8) |

[a]Test group vs tired patients: t-test, 143 d.f., $P<0.001$. [b]Test group vs not tired patients: t-test, 154 d.f., $P=0.0003$.

score of ⩽9 on RMBT. Profile scores for those tested showed 45 patients (49%) with poor memory and 24 (26%) with moderate impairment. One patient was found to have severe memory impairment.

*Comparison of subjective complaints and performance on objective testing*

Those who rated their concentration and/or memory difficulties 3 or 4, i.e. 'quite a bit' or 'very much' on those items (Q20 and Q25) of the EORTC QLQ-C30, were designated 'complainers' for the purposes of this study.

Subjective complaints of concentration and memory were not related to IQ as estimated by the NART. Median IQ estimates for complainers and non-complainers of concentration difficulties were 124 and 118 respectively (Mann-Whitney $z=1.6$, $P=0.1$). Median IQ estimates for complainers and non-complainers of memory problems were 123 and 122 (Mann-Whitney $z=0.5$, $P=0.6$) respectively. Scores on the cognitive functioning scale were not correlated with NART scores ($r_s=0.005$, $n=91$, $P=1.0$).

PASAT scores for those who complained of concentration difficulties (median 22, range 0-49, $n=11$) were not significantly different (Mann-Whitney $z=0.2$, $P=0.8$) from whose who did not report concentration difficulties (median 24, range 0-47, $n=80$).

There were no significant differences betwee those who did, and did not, complain of memory problems in performance on RMBT. The median profile score for complainers was 18 (range 14-24, $n=14$) and for non-complainers was 19 (range 9-24, $n=77$); Mann-Whitney $z=0.6$, $P=0.5$). The median screening scores were 8.5 (range 5-12, $n=14$) and 9 (range 3-12, $n=77$; Mann-Whitney $z=0.2$, $P=0.9$) respectively.

Scores on the EORTC cognitive functioning scale were not correlated with scores on PASAT ($r_s=0.5$, $n=88$, $P=0.7$) or on RMBT (profile: $r_s=0.07$, $n=91$, $P=0.5$; screening: $r_s=0.01$, $n=91$, $P=0.9$).

*The relationship of subjective complaints of cognitive dysfunction to anxiety, depression and fatigue*

The significant differences in scores on anxiety, depression and fatigue scales between those who did and did not complain of concentration and memory difficulties are shown in Table III.

Scores on the EORTC QLQ-C30 cognitive functioning scale were highly correlated with HADS anxiety and depression scores, in each case $r_s=0.5$, $P<0.0001$. Cognitive functioning scores were significantly correlated with all self-report scales of fatigue (i.e. MFI general fatigue, $r_s=0.5$, $P<0.0001$; MFI physical fatigue, $r_s=0.3$, $P=0.007$; MFI reduced activity, $r_s=0.2$, $P=0.03$; MFI mental fatigue, $r_s=0.7$, $P<0.0001$; MFI reduced motivation, $r_s=0.4$, $P=0.0003$; BFMQ, $r_s=0.7$, $P<0.0001$).

*The use of strategies to improve memory*

Those who complained of concentration difficulties on the EORTC QLQ-C30 used significantly more external memory

**Table III** Comparison of mean anxiety, depression and fatigue scores for those who do and do not complain of concentration and memory difficulties

| | *Q20 concentration* Non-complainers (n=80) | *Q20 concentration* Complainers[a] (n=11) | *Q25 memory* Non-complainers (n=77) | *Q25 memory* Complainers[a] (n=14) |
|---|---|---|---|---|
| HAD anxiety | | | | |
| Mean score | 4.9 (3.7) | 10.1 (5.7)*** | 4.7 (3.5) | 10.4 (5.2)*** |
| Percentage of cases | 23 | 64 | 19 | 71 |
| HAD depression | | | | |
| Mean scores (s.d.) | 3.2 (2.7) | 6.8 (3.4)*** | 2.9 (2.5) | 7.4 (3.1)*** |
| Percentage of cases | 8 | 45 | 5 | 50 |
| HAD (A+D) | | | | |
| Mean score (s.d.) | 8.1 (5.6) | 16.9 (8.6)*** | 7.6 (5.1) | 17.9 (7.6)*** |
| Percentage of cases>19 | 9 | 45 | 5 | 57 |
| Mean MFI (s.d.) | | | | |
| General fatigue | 10.1 (3.8) | 13.8** (3.1) | 9.9 (3.7) | 14.1*** (2.8) |
| Physical fatigue | 9.7 (4.0) | 10.7 (3.8) | 9.5 (4.0) | 11.7* (3.5) |
| Reduced activity | 8.6 (4.0) | 10.3 (4.1) | 8.4 (3.8) | 10.9* (4.3) |
| Mental fatigue | 7.4 (3.6) | 15.5*** (3.1) | 7.1 (3.4) | 15.2*** (3.2) |
| Reduced motivation | 7.6 (3.3) | 9.8* (3.7) | 7.5 (3.2) | 9.9* (4.0) |
| BMFQ mean (s.d.) | 5.2 (5.0) | 20.7 (5.3)*** | 4.9 (4.5) | 19.3 (6.7)*** |

[a]Q20/Q25 of EORTC QLQ-C30 rated 3-4 (quite a bit to very much). *$P<0.05$, **$P<0.01$, ***$P<0.001$.

**Table IV** Regression analyses of subjective complaints of cognitive impairment

(a) Logistic regression-EORTC concentration/memory item scores

| | *Odds ratio* | *95% confidence intervals* | P-*value* |
|---|---|---|---|
| Concentration | | | |
| MFI mental fatigue | 1.7 | 1.3-2.3 | 0.001 |
| HAD depression | 6.7 | 0.8-54.0 | 0.07 |
| Memory | | | |
| MFI mental fatigue | 2.1 | 1.3-3.4 | 0.001 |
| HAD depression | 52 | 3.1-873.0 | 0.006 |

(b) Multiple Regression-EORTC QLQ-C30 cognitive functioning (CF) Scale

CF score = 95.5 + 18.7 (HAD 'case' depression) − 3.7 (MFI mental fatigue score).

aids, e.g. lists (median 5, range 2–7; $z=3.4$, $P=0.0006$), and more internal strategies, e.g. alphabetical searching, mental retracing (median 3, range 1–6; $z=2.6$, $P=0.008$), than non-complainers (external aids: median 3, range 0–6; internal strategies: median 2, range 0–5).

Similarly, those who complained of memory difficulties reported using more external aids (median 4, range 2–6; $z=3.2$, $P=0.001$) and internal strategies (median 3, range 1–6; $z=2.71$, $P=0.007$) than non-complainers (external aids: median 3, range 0–7; internal strategies median 2, range 0–5).

Scores on the cognitive functioning scale were correlated with the number of external aids ($r_s=0.49$, $n=91$, $P<0.0001$) and internal strategies ($r_s=0.32$, $n=91$, $P=0.002$) used to assist memory.

*Cognitive function and quality of life*

Complainers and non-complainers of concentration difficulties showed no significant differences in ratings of their health or global QL on the EORTC QLQ-C30 global health status/QL scale. By contrast, those who complained of memory problems did have lower scores on the global health status/QL scale (Mann–Whitney $z=2.4$, $P=0.02$). This difference was attributable to the complainers' lower rating of their overall health (Mann–Whitney $z=2.6$, $P=0.01$). For the samples as a whole, scores on the cognitive functioning scale were correlated with scores on the global health status/QL scale ($r_s=0.3$, $n=91$, $P=0.007$).

*Who are the complainers?*

Univariate analysis showed no significant differences between complainers and non-complainers of concentration or memory difficulties in sociodemographic characteristics (age, sex, employment status, estimated premorbid IQ or educational level) or in their clinical characteristics (diagnosis, chemotherapy or not, intensity/duration of treatment, time since treatment stopped).

Regression analysis was therefore carried out using the HADS and fatigue scale scores to predict subjective complaints of concentration and memory difficulties. The MFI mental fatigue scale and 'caseness' on the HADS depression scale independently predicted complaints of concentration and memory difficulties and scores on the EORTC cognitive functioning scale (Table IV).

### Discussion

The EORTC QLQ-C30 is increasingly widely used internationally as an outcome measure in cancer clinical trials. Its cognitive functioning scale has not until now been validated against objective test data. It is, therefore, not clear what subjective complaints of concentration or memory difficulties derived from this scale actually mean.

Cancer patients' cognitive function may be compromised by organic impairment attributable to the disease process or treatment. These effects may be temporary and reversible; however, if the cognitive dysfunction complained of is associated with persistent difficulties in everyday living, this information deserves greater prominence in the reporting of treatment outcomes. On the other hand, complaints of cognitive difficulties may reflect psychological distress or fatigue, which are recognised as common among cancer patients. This distinction has important implications for the type of intervention that should be offered. Techniques may be taught to try to limit the problems caused by organic memory impairment, e.g. use of external memory aids, but mood disturbance is amenable to potentially curative intervention and warrants direct attention.

Patients treated for Hodgkin's disease and many patients with non-Hodgkin's lymphoma have an excellent prognosis in terms of survival, but the quality of their functional recovery has been called into question (van Tulder, 1994; Razavi *et al*., 1993), and cognitive dysfunction may be a relevant factor. This patient population, therefore, offers the opportunity to examine the relationship between subjective complaints of concentration and memory difficulties and performance on objective testing in a setting in which the impact of active disease process and transient pharmacological effects can be excluded and the issue has clinical significance for patients' quality of life.

Difficulties were anticipated in tracing and recruiting long-term cancer survivors for a hospital-based follow-up study. We were greatly helped by access to the Scotland and Newcastle Lymphoma Group Register. The response rate was excellent, with only 12% of those contacted declining to participate. The reasons given ranged from 'feeling too well' to 'not feeling well enough', suggesting no systematic sampling bias in our data. Subjective complaints of concentration and/or memory difficulties were more common among those agreeing to undergo testing, and although the low prevalence of complaints was clinically encouraging this meant that the study had low power to detect differences between those who did and did not complain. Replication in a larger sample is needed.

The mean estimated IQ of those tested was relatively high, but there was no evidence that the estimated level of intellectual ability was associated with subjective complaints of concentration or memory difficulties.

The objective testing procedure was carefully chosen to be brief and user friendly, to encourage participation and yet to be sufficiently sensitive to act as a screen for organic impairment.

Subjective complaints of memory problems were compared with performance on the RBMT, which was specifically designed to reflect memory skills used in daily life. Scores on RBMT relate significantly to conventional memory tests but correlate better with patients' and relatives' reports of dysfunction than conventional measures (Lincoln and Tinson, 1989). Subjective complaints of concentration difficulties were compared with performance on the PASAT. This test, which requires divided attention, sustained concentration and efficient information processing, has been shown to be sensitive to subtle neurocognitive deficits. In other patient samples PASAT scores have been found to mirror patients' self-reported difficulties (Johnson *et al*., 1994). Patients may have difficulty in distinguishing problems with information processing/encoding from problems with information storage or retrieval from memory and, to allow for this, scores on the EORTC cognitive function scale as a whole were compared with performance on both tests. No relationship could be demonstrated in any of these analyses between patients' self-reported difficulties and their performance on objective testing. The interpretation of the meaning of this two-item scale is therefore open to question. It may be that, to obtain self-report data about cognitive function that are related to observable performance, more detailed questions will have to be asked.

Those who complained of difficulties with concentration or memory were significantly more likely to be clinically anxious or depressed. Of this sample, 10% were identified as probable cases warranting at least further assessment of their distress. Complainers of memory problems were more likely to be identified as probable cases than non-complainers. Evidence from other patient populations relates both anxiety and depression to increased memory complaints. Those scoring $\geqslant 8$ on the HADS depression scale were significantly more likely to complain of concentration and, particularly, memory difficulties. These data suggest that patients in remission who report memory or concentration difficulties rated 3 or 4 on the EORTC scale should be screened for clinically significant anxiety and/or depression. Effective intervention to relieve affective disorder might be expected to be accompanied by improvement in subjectively reported cognitive difficulties.

Subjective reports of concentration and memory difficulties, which were associated with higher fatigue scores and multivariate analysis, showed the MFI mental fatigue scale, in particular, to be an independent predictor of subjective complaints on the EORTC items. Responses to the EORTC cognitive functioning scale may, then, be interpreted in terms of mental fatigue, but current understanding of the basis of mental fatigue leaves the clinical significance of this observation in some doubt. There is a generally high correlation between fatigue scale scores and measures of anxiety and depression, observable both in this sample and in the literature (Fobair *et al.*, 1986; Smets *et al.*, 1996). Whether or not some common underlying physiological mechanism can be elucidated, this finding further underlines the importance of assessing patients who complain of cognitive dysfunction or fatigue for clinically significant and potentially remediable emotional disturbance.

Contrary to expectations, complainers of concentration and memory difficulties reported using significantly more aids to memory, both external aids, e.g. lists, and internal strategies, e.g. mental retracing, than non-complainers. Having to use these aids may be viewed as an indicator of cognitive failure resulting in the observed self-reports of difficulties. Clinically, it would be wise to check whether these coping strategies are being used efficiently, but the primary action to relieve subjectively experienced problems in patients with no objective evidence of impairment focuses on addressing anxiety/depression.

As expected, those who reported better cognitive functioning also reported a better global quality of life.

On objective testing, this sample exhibited evidence of cognitive dysfunction in that the mean scores on the PASAT were lower than reference data from an older sample. In addition, 63% of the sample were identified by the RBMT screening score as memory impaired. These findings warrant further exploration.

The key point to emerge from this study is that what lymphoma patients in remission mean when they complain of concentration or memory problems on the EORTC QLQ-C30 cannot be measured on well-validated objective measures of concentration or memory. Their subjective reports appear to reflect affective disorder and mental fatigue. While this finding needs to be replicated in a larger sample of patients with complaints about their cognitive function and among those with different disease sites, treatment histories etc., it does call into question the validity of such a brief cognitive function scale.

Validity may be improved by the inclusion of additional items about specific aspects of cognitive function exemplified in activities of daily living. In other settings, it has been suggested that patients' subjective reports of their cognitive function should be collected in diary format and that relatives'/carers' reports are more reliable (Herrmann, 1982). Until this can be demonstrated satisfactorily, objective performance testing remains the method of choice for assessing higher mental function.

**Acknowledgements**
The authors would like to thank Dr L Matheson and Dr RCF Leonard for permission to include their patients, Lynda Mill for her invaluable help in tracing patients and Mrs E Traynor for administrative assistance.

### References

AARONSON NK, AHMEDZAI S, BERGMAN B, BULLINGER M, CULL A, DUEZ NJ, FILIBERTI A, FLECHTNER H, FLEISHMAN SB, DE HAES JCJM, KAASA S, KLEE M, OSOBA D, RAZAVI D, ROFE PB, SCHRAUB S, SNEEUW K, SULLIVAN M AND TAKEDA F. FOR THE EUROPEAN ORGANIZATION FOR RESEARCH AND TREATMENT OF CANCER STUDY GROUP ON QUALITY OF LIFE. (1993). The EORTC QLQ-C30: a quality of life instrument for use in international clinical trials in oncology. *J. Natl Cancer Inst.*, **85**, 365–376.

BENTALL RP, WOOD GC, MARRINAN T, DEANS C AND EDWARDS RHT. (1993). A brief mental fatigue questionnaire. *Br. J. Clin. Psychol.*, **32**, 375–377.

CORCORAN R AND THOMSON P. (1993). Epilepsy and poor memory: who complains and what do they mean? *Br. J. Clin. Psychol.*, **32**, 199–208.

CULL A, STEWART M AND ALTMAN DG. (1995). Assessment of and intervention for psychosocial problems in routine oncology practice. *Br. J. Cancer*, **72**, 229–235.

DEVLEN J, MAGUIRE P, PHILLIPS P AND CROWTHER D. (1987). Psychological problems associated with diagnosis and treatment of lymphomas II. Prospective study. *Br. Med. J.*, **295**, 955–957.

FOBAIR P, HOPPE RT, BLOOM J, COX R, VAUGHESE A AND SPIEGEL D. (1986). Psychological problems among survivors of Hodgkin's disease. *J. Clin. Oncol.*, **4**, 805–814.

GRONWALL DMA. (1977). Paced auditory serial addition task. A measure of recovery from concussion. *Perc. Motor Skills*, **44**, 367–373.

GRONWALL DMA AND SAMPSON H. (1974). *The Psychological Effects of Concussion.* Auckland University Press/Oxford University Press: New Zealand.

DE HAES JCJM, VAN KNIPPENBERG FCE AND NEIJT JP. (1990). Measuring psychological and physical distress in cancer patients: structure and application of the Rotterdam Symptom Checklist. *Br. J. Cancer*, **62**, 1034–1038.

HERMANN DJ. (1982). Know thy memory: the use of questionnaires to assess and study memory. *Psychol. Bull.*, **92**, 434–452.

IBBOTSON T, MAGUIRE P, SELBY P, PRIESTMAN T AND WALLACE L. (1994). Screening for anxiety and depression in cancer patients: the effects of disease and treatment. *Eur. J. Cancer*, **30A**, 37–40.

JOHNSON SK, DE LUCA JD, FIEDLER N AND NATELSON BH. (1994). Cognitive functioning of patients with Chronic Fatigue Syndrome. *Clin. Infect. Dis.*, **18**, **(Suppl)**, 584–585.

LINCOLN NB AND TINSON R. (1989). The relationship between subjective and objective memory impairment after stroke. *Br. J. Clin. Psychol.*, **28**, 61–65.

MCDONALD E, COPE H AND DAVID A. (1993). Do laboratory tests predict everyday memory? A neuropsychological study. *J. Verb. Learn. Verb. Behav.*, **22**, 341–357.

NELSON HE AND WILLISON JR. (1991). *The National Adult Reading Test* 2nd edn. *Test Manual.* NFER-Nelson: Windsor.

RAZAVI D, DELVAUX N, BREDART A, AUTIER P, BRON D, DENUSSCHER L AND STRYCHMANS P. (1993). Professional rehabilitation of lymphoma patients. A study of psychosocial factors associated with return to work. *Supp. Care Cancer*, **1**, 276–278.

ROMAN DD, EDWALL GE, BUCHANAN RJ AND PATTON JH. (1991). Extended norms for the paced auditory serial addition task. *Clin. Neuropsychol.*, **5**, 33–40.

SMETS EMA, GARSSEN B, SCHUSTER UITTERHEOVE ALJ AND DE HAES JCJM. (1993). Fatigue in cancer patients. *Br. J. Cancer*, **68**, 220–224.

SMETS EMA, GARSSEN B, BONKE B AND DE HAES JCJM. (1995). The multidimensional fatigue inventory: psychometric qualities of an instrument to assess fatigue. *J. Psychosom. Res.*, **39**, 315–325.

SMETS EMA, GARSSEN B, CULL A AND DE HAES JCJM. (1996). Application of the multidimensional fatigue inventory in cancer patients receiving radiotherapy. *Br. J. Cancer*, **73**, 241–245.

SUNDERLAND A, HAMS JE AND BADDELEY AD. (1983). Do laboratory tests predict everyday memory? A neuropsychological study. *J. Verb. Learn. Verb. Behav.*, **22**, 341–357.

VAN TULDER MW, AARONSON NK AND BRUNING PF. (1994). The quality of long term survivors of Hodgkin's disease. *Ann. Oncol.*, **5**, 153–158.

WILSON B, COCKBURN J AND BADDLEY A. (1991). *The Rivermead Behavioural Memory Test*, 2nd edn. *Test Manual.* Thames Valley Test Company: Bury St. Edmonds.

ZIGMOND AS AND SNAITH RP. (1983). The Hospital Anxiety and Depression Scale. *Acta Psychiatr. Scand.*, **67**, 361–370.